# Exploring the medical decision-making patterns and influencing factors among the general Chinese public: a binary logistic regression analysis

**DOI:** 10.1186/s12889-024-18338-8

**Published:** 2024-03-25

**Authors:** Yuwen Lyu, Qian Xu, Junrong Liu

**Affiliations:** 1 Institute of Humanities and Social Sciences, Guangzhou Medical University, Guangzhou, 511436 China; 2 School of Health Management, Guangzhou Medical University, Guangzhou, 511436 China

**Keywords:** Medical decision, Decision-making patterns, Influencing factors, Logistic regression

## Abstract

**Objective:**

With the ongoing evolution of the healthcare system and shifts in cultural paradigms, there is a pressing need to delve into the medical decision-making behaviors of general Chinese public and understand their underlying motivations. This research seeks to elucidate the prevailing tendencies in these decision-making processes and to empirically validate the pivotal factors that shape their choices, offering valuable insights for healthcare policymakers and institutions.

**Method:**

A comprehensive survey was administered to 2,696 Chinese residents to examine their medical decision-making patterns. These patterns were classified into two primary categories: Unilateral Decision-making (Doctor-dominant, Family-centric, and Patient-driven subtypes) and Collaborative Decision-making (Doctor-led, Doctor-Patient, Patient-Family, and Doctor-Patient-Family subtypes). Binary logistic regression analysis was employed to empirically pinpoint the significant factors influencing these decision-making frameworks.

**Results:**

The study's analysis reveals distinct preferences in medical decision-making among Chinese residents. In the Collaborative Decision-making category, chosen by 70.81% of participants, the subtypes are as follows: Doctor-led (29.90%), Doctor-Patient (13.54%), Patient-Family (2.93%), and Doctor-Patient-Family (24.44%). The Unilateral Decision-making, preferred by 29.19%, includes Doctor-dominant (23.22%), Family-centric (1.74%), and Patient-driven (4.23%) models. The preference for Collaborative Decision-making is associated with higher educational levels, specific marital statuses (particularly married but childless), and choices of rural residents' basic medical insurance or occupational basic medical insurance. In contrast, Unilateral Decision-making correlates with males, individuals with religious beliefs, certain occupational roles (like civil servants), and holders of commercial or publicly funded medical insurance.

**Conclusion:**

This study elucidates the complex interplay of socio-cultural and individual determinants shaping medical decision-making in China. The findings reveal a marked inclination towards collaborative models, closely linked to educational level, marital status, and specific insurance types, reflecting an evolving trend towards participatory healthcare. Simultaneously, the persistence of unilateral models, influenced by gender, religious beliefs, and occupational roles, highlights the heterogeneity within Chinese healthcare preferences. These insights are crucial for policymakers and healthcare practitioners, underscoring the need for adaptable, culturally attuned healthcare frameworks that cater to this diversity, thereby enhancing patient engagement and healthcare efficacy.

**Supplementary Information:**

The online version contains supplementary material available at 10.1186/s12889-024-18338-8.

## Introduction

Medical decision-making encompasses the deliberative process of choosing the most efficacious diagnostic and therapeutic options from a range of alternatives. Central to this process are stakeholders such as physicians, patients, and their families [41]. Given the disparate interests and value perspectives among these stakeholders, a spectrum of medical decision-making models has been delineated. Contemporary research posits that, based on the dynamics of communication and the power equilibrium between physicians and patients, these models can be broadly segmented into two primary paradigms: Unilateral Decision-making and Collaborative Decision-making [8, 18].

Unilateral decision-making denotes a paradigm wherein a singular entity possesses the definitive authority in the medical decision-making process. This model can be further segmented into the doctor-dominant, family-centric, and patient-driven subtypes. In the doctor-dominant subtype, physicians, leveraging their expertise and experiential knowledge, independently chart the course of medical action. However, this approach often relegates patients and their families to subordinate roles, primarily involving them in information provision and adherence to prescribed treatment regimens. Such a paradigm can potentially undermine patient autonomy and hinder the advancement of informed consent processes, thereby risking suboptimal therapeutic outcomes [24]. In the family-centric subtype of medical decision-making, the patient's relatives, upon considering the physician's advice, make choices that align with their understanding of the patient's health and their core values. On the other hand, the patient-driven subtype is exemplified by patients independently directing their medical journey, guided by their personal values and preferences. Historically in Chinese medicine, the family has played a pivotal role in patient healthcare activities, exerting a significant authoritative influence in healthcare decision-making processes [12].

The traditional model of Unilateral decision-making is increasingly seen as inadequate in the context of contemporary medical practice, paving the way for the emergence of the collaborative decision-making model [49]. Collaborative Decision-making is a paradigm where multiple stakeholders actively participate and reach a consensus during the medical process. This model can be further delineated into the doctor-led, doctor-patient, patient-family, and doctor -patient-family [29]. In the doctor-led subtype, the physician, while considering the patient's input, primarily guides the treatment direction. However, the heart of Collaborative Decision-making lies in the doctor-patient model, a true embodiment of Shared Decision Making (SDM). Here, the physician and patient engage in an egalitarian dialogue, blending medical expertise with the patient's values to jointly formulate a treatment plan. This collaborative approach aligns with SDM frameworks like Elwyn et al.'s "three-talk model" and the Ottawa Decision Support Framework, which focus on structured decision-making phases and informed deliberation [10, 42]. The significance of such collaborative models, especially SDM, is underscored by research from Pieterse AH et al. [16, 38]. These studies highlight the crucial role of patient-physician collaboration and explore the challenges and facilitators encountered in implementing SDM across varied healthcare settings. The patient-family model adds another layer, involving both the patient and their family in decision-making, ensuring that treatment plans reflect both the patient's and family's values and perspectives. Lastly, the tripartite collaboration model, involving physicians, patients, and families, aligns with Chinese cultural values [11]. It emphasizes collective decision-making and respectful communication, reflecting the communal and family-oriented nature of Chinese society.

Each medical decision-making model possesses its inherent characteristics, underscoring the nuanced roles, power dynamics, and responsibilities of the involved stakeholders [35, 50]. A conceptual framework, based on existing literature, classifies these models into two broad categories: Unilateral Decision-making and Collaborative Decision-making [29].

Unilateral Decision-making encompasses three subtypes:

- Doctor-Dominant: The physician primarily makes decisions, often with limited patient or family input.
- Family-Centric: Decision-making is led by family members, who consider the patient's needs and the physician's advice.
- Patient-Driven: The patient independently makes decisions, possibly informed by their research or personal preferences.

Collaborative Decision-making, in contrast, involves multiple stakeholders and includes:

- Doctor-Led: The physician guides the decision process but incorporates patient input.
- Doctor-Patient: An egalitarian model where physician and patient jointly make decisions, embodying the essence of Shared Decision Making (SDM).
- Patient-Family: Decisions are made collaboratively between the patient and family members.
- Doctor-Patient-Family: This model integrates the physician, patient, and family in a tripartite decision-making process.

The choice of a model is influenced by factors related to both physicians and patients, such as demographics professional standing, health status, cultural background, and the doctor-patient relationship. Research across various cultures has provided insights into these dynamics [4, 15]. In Italy, physicians' personal beliefs and specialties influence their decisions in early breast cancer treatment, underscoring the impact of individual professional judgment [28]. Simultaneously, the study found that Bengali patients received more supportive communication from Bengali doctors than ethnic minority patients, underscoring the importance of patient-centered communication for equitable healthcare across ethnicities [54]. Moreover, the study emphasized the complexity of patient values in decision-making, revealing that individuals with type 2 diabetes consider not just treatment-specific factors, but also life goals, philosophies, and personal and social backgrounds when deciding about insulin treatment, suggesting a need for a broader understanding of patient values in clinical decision-making [25].

In this context, a study from China contributes further by analyzing preferences in medical decision-making models among different demographics, revealing significant variations based on gender, age, and education level. This research indicates a general preference for physician-led decision-making, particularly the directive-collaborative model [29]. However, it primarily focuses on statistical preferences and correlations, pointing to a gap in the comprehensive understanding of specific factors influencing these preferences in the Chinese healthcare context. Building on previous research, this study is designed to address the scarcity of research in China on the diverse factors influencing medical decision-making, which has broader implications on a global scale. It aims to dissect public preferences and the array of factors impacting these choices in a Chinese context, providing insights that can be valuable not only within China but also for international comparisons. By systematically examining variables such as individual, familial, occupational, and regional characteristics, along with medical insurance types, it contributes to the global understanding of how these elements shape healthcare decision processes. It seeks to enhance global understanding of healthcare decision processes, thus advancing patient-centered medical practices in various cultural and healthcare systems.

## Method

### Binary logistic regression

The binary logistic model, a specific variant of the broader multinomial logistic models, stands as a sophisticated statistical instrument adept at elucidating intricate interrelationships among observed variables, factoring in multifaceted interactions and influences. Its utility in the medical sphere is well-established. For instance, it has been leveraged to evaluate the interplay between individual attributes, such as age, gender, and genetic markers, and their association with risks tied to diverse chronic ailments [44]. Additionally, these models have been pivotal in shedding light on the nexus between environmental determinants, lifestyle choices, and other health-centric behaviors and a spectrum of health outcomes [39]. In the context of healthcare service consumption, multinomial logistic models have been tapped to dissect the factors underpinning patients' predilections for distinct medical services. For example, scholars have employed this paradigm to discern the influence of variables like household financial standing, health insurance coverage, and geographical positioning on patients' proclivities towards outpatient, inpatient, or emergency services [34]. Therefore, the deployment of the binary logistic model to analyze propensities in medical decision-making and their pertinent drivers can illuminate the inherent dynamics among these variables. Such insights can offer a profound understanding of the cognitive and behavioral tendencies individuals manifest when navigating medical decisions. This enriched perspective not only equips healthcare professionals to more adeptly cater to patients' aspirations and anticipations, thereby elevating healthcare service caliber but also enables patients to gain a nuanced grasp of their medical scenarios and treatment alternatives, promoting a more informed and engaged role in their healthcare decision-making process.

Binary Logistic Regression is primarily employed for a dependent variable with two categories. In this study, the dependent variable is specifically categorized into "Unilateral Decision-making" and "Collaborative Decision-making." Here, "Unilateral Decision-making" is designated as the dummy variable (with a value of 1), while "Collaborative Decision-making" serves as the reference category (assigned a value of 0). The form [23] of the model is:

$${\text{log}}\left(\frac{p}{1-p}\right)={\beta }_{0}+{\beta }_{1}{X}_{1}+{\beta }_{2}{X}_{2}+\dots +{\beta }_{k}{X}_{k}$$

Where $p$ represents the probability of the event occurring, ${X}_{1},{X}_{2},\dots ,{X}_{k}$ is the independent variable, and ${\beta }_{0},{\beta }_{1},\dots ,{\beta }_{k}$ is the regression coefficient.

To assess the goodness of fit and explanatory power of the model, this study employs the McFadden R-squared statistical metric. This metric offers a quantified approach to gauge the extent to which the model fits the observed data, thereby ensuring the model's reliability and accuracy.

### Variable selection and measurement

The sample data originates from a national-level research project completed by our research group in 2022, was collected from a broad spectrum of the general Chinese public. To ensure the quality and clarity of the survey, and to avoid ambiguity in responses, the concept of medical decision-making was clearly defined within the questionnaire. The survey was custom-designed to align with the research objectives and content and then integrated into the Questionnaire Star enterprise system. After a preliminary survey and consultations with experts in statistics and medical ethics, the questionnaire underwent three rounds of revisions, resulting in the final version that is detailed in Appendix 1.

Participants in the sample ranged in age from 18 to over 75 years old, with educational backgrounds spanning from junior high school or below to undergraduate level and above. Additionally, data was collected on participants' occupational backgrounds, monthly family income, medical payment methods, religious beliefs, and family circumstances, among other relevant factors. A detailed description of the sample and variable definitions can be found in Table 1.

**Table 1:** Sample description and variable definitions

Variable Name	Definition	Value Range/Encoding
Gender	Participant's Gender	Male=1, Female=0
Age	Age Group of the Participant	18-44 years old=1, 45-59 years old=2, 60-74 years old=3, 75 years old and above=4
Education	Highest Educational Level of the Participant	Junior High School and below=1, Associate Degree=2, Bachelor's Degree and above=3
Occupation	Occupation Category of the Participant	Civil Servant=1, Corporate Staff=2, Enterprise Worker=3, Self-employed=4, Migrant Worker=5, Retiree=6, Freelancer=7, Public Institution Employee=8, Medical Institution Staff=9, Unemployed=10
Monthly Family Income	Monthly Income Range of the Participant's Family	Below 5,000 yuan (≈ Below $775) = 1, 5,000-8,000 yuan (≈ $775-$1,240) = 2, 8,000-12,000 yuan (≈ $1,240-$1,860) = 3, 12,000-23,000 yuan (≈ $1,860-$3,565) = 4, Above 23,000 yuan (≈ Above $3,565) = 5
Medical Payment Method	Participant's Method of Medical Payment	Fully Self-Paid = 1, Publicly Funded Medical Care = 2, Commercial Insurance = 3, Occupational Basic Medical Insurance = 4, Urban Residents' Basic Medical Insurance = 5, Rural Residents' Basic Medical Insurance = 6
Religious Belief	Whether the Participant has a Religious Belief	Yes = 1, No = 0
Family Situation	Participant's Family Structure	Unmarried = 1, Married without Children = 2, Widowed/Divorced with Children = 3, Children studying or working away, couple living together = 4, Couple living with children = 5, Elderly-centered, two or more couples and their children living together = 6, Grandparents living with grandchildren = 7, Other family types = 8
Region	Geographic Area where the Participant Resides	Eastern = 1, Central = 2, Western = 3 (Note: For regional divisions, see Appendix 1)
Medical Decision-making Model	Preferred Medical Decision-making Model by the Participant	Doctor-dominant = 1, Family-centric = 2, Patient-drivenl = 3, Doctor-led = 4, Doctor-Patient = 5, Patient-Family = 6, Doctor-Patient-Family = 7

### Data collection and sampling

#### Survey methodology and quality assurance

The data collection methodology for this study, utilized the Questionnaire Star system, a professional online survey tool, to ensure the accuracy and representativeness of the data gathered from participants. A stratified convenience sampling technique was employed to collect responses from a diverse sample of 2,696 individuals, spanning 31 provinces and cities across China, with the exclusion of Hong Kong, Macao, and Taiwan regions. To ensure the validity of the responses, the Questionnaire Star system was programmed with quality control measures such as quota rules for different occupational groups, time requirements to discourage incomplete responses, and anti-duplication measures like one-time entries per IP address, computer, or mobile device.

#### Sample size determination and statistical power

The survey was disseminated through WeChat, facilitating convenient access for participants and resulting in a substantial response rate of 48.54%, after the exclusion of 2,858 invalid responses through automated isolation techniques. The robustness of the sampling process was further ensured by a power analysis conducted using G*Power 3.1.9.7 [13], which determined that a sample size of 2040 was required to achieve a power of 0.95, given the medium effect size (OR = 1.2) and alpha level of 0.05. The actual power achieved post hoc was 0.9507, indicating that the study was sufficiently powered to detect the specified effect size. This comprehensive approach to data collection and sampling underscores the study's commitment to rigorous research standards and provides a strong foundation for the subsequent analysis of medical decision-making patterns among the general Chinese public.

## Results

### Descriptive statistical analysis

To gain a comprehensive understanding of the demographic attributes of the sample population, an in-depth analysis was carried out. The sample encompasses a wide array of individuals, representing various ages, genders, educational backgrounds, occupations, income levels, family situations, living arrangements, and religious beliefs. This detailed demographic profiling is essential for contextualizing the research findings and assessing the representativeness of the sample. Presented below in Table 2 is a summary of these demographic and sociological characteristics.

**Table 2:** Demographic and sociological characteristics of the sample population

Variable	Option	Frequency (n)	Percentage (%)
Gender	Male	1052	39.0
	Female	1644	61.0
Age	18-44	2253	83.6
	45-59	408	15.1
	60-74	32	1.2
	75 and above	3	0.1
Education Level	Junior High School and below	753	27.9
	Associate Degree	584	21.7
	Bachelor's Degree and above	1359	50.4
Occupation	Civil Servant	250	9.3
	Corporate Staff	502	18.6
	Enterprise Worker	265	9.8
	Self-employe	181	6.7
	Migrant Worke	85	3.2
	Retiree	31	1.1
	Freelancer	227	8.4
	Public Institution Employe	339	12.6
	Medical Institution Staff	650	24.1
	Unemployed	166	6.2
Monthly Family Income	Below 5,000 yuan (≈ Below $775)	744	27.6
	5,000-8,000 yuan (≈ $775-$1,240)	769	28.5
	8,000-12,000 yuan (≈ $1,240-$1,860)	665	24.7
	12,000-23,000 yuan (≈ $1,860-$3,565)	361	13.4
	Above 23,000 yuan (≈ Above $3,565)	157	5.8
Medical Payment Method	Fully Self-Paid	595	22.1%
	Publicly Funded Medical Care	845	31.3%
	Commercial Insurance	555	20.6%
	Occupational Basic Medical Insurance	1130	41.9%
	Urban Residents' Basic Medical Insurance	659	24.4%
	Rural Residents' Basic Medical Insurance	505	18.7%
Religious Belief	Yes	554	20.5
	No (Atheist)	2142	79.5
Family Situation	Unmarried	771	28.6
	Married without Children	610	22.6
	Widowed/Divorced with Children	70	2.6
	Children studying or working away, couple living together	236	8.8
	Couple living with children	813	30.2
	Elderly-centered, two or more couples and their children living together	106	3.9
	Grandparents living with grandchildren	66	2.4
	Other family types	24	0.9
Total		2696	100.0

This study conducted a detailed classification and statistical analysis of the medical decision-making models of 2,696 Chinese residents. The specific results can be found in Table 3.

**Table 3:** Selection outcomes for medical decision-making model

Pattern	Classification	Subtype	Frequency	Percentage(%)
Decision-making pattern	Unilateral Decision-making	Doctor-dominant	626	23.22
		Family-centric	47	1.74
		Patient-driven	114	4.23
		Total	787	29.19
	Collaborative Decision-making	Doctor-led	806	29.90
		Doctor-Patient	365	13.54
		Patient-Family	79	2.93
		Doctor-Patient-Family	659	24.44
		Total	1909	70.81
Total			2696	100.0

**Fig. 1:**
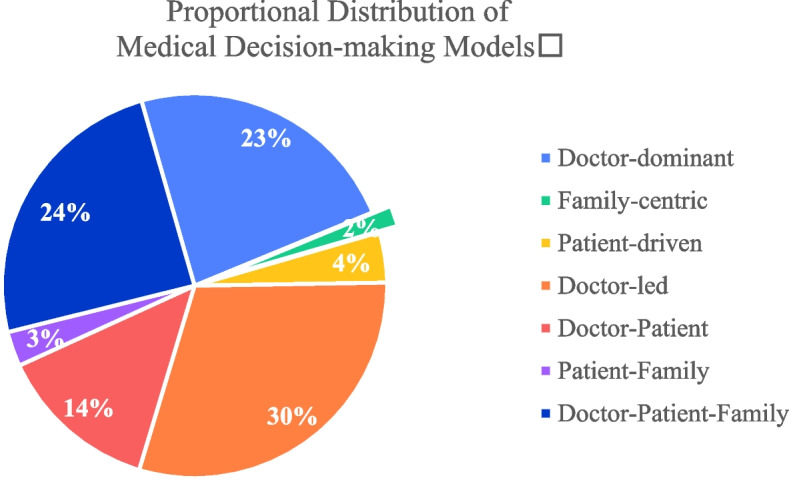
Distribution Pie Chart of Each Decision-making Model

According to the survey results presented in Table 3 and Fig. 1, only 29.19% of Chinese residents opt for the Unilateral decision-making model, while a staggering 70.81% prefer the collaborative decision-making model. This underscores the primary trend in China's medical decision-making, which is gradually shifting towards doctor-patient collaboration , rather than sole autonomous decision-making.

First and foremost, within the realm of collaborative decision-making, the doctor-led collaborative model emerges as predominant, constituting 29.90% of the choices. This underscores the pivotal role physicians play in the decision-making trajectory, with patients actively engaging in the process, albeit under the physician's guidance. Additionally, the model where both the doctor-patient jointly partake in decision-making is also relatively prevalent, accounting for 13.54%. In the Chinese context, the decision-making model involving both the patient and their family members accounts for 2.93%, while the tripartite model encompassing the physician, patient, and family members constitutes 24.44%.

Secondly, within the realm of unilateral decision-making, the doctor-dominant model emerges as predominant, accounting for 23.22%. This reflects the elevated authoritative status bestowed upon doctors in medical settings due to their specialized knowledge and experience. However, the proportions of singular decision-making models led by family members and patients are relatively low, standing at 1.74% and 4.23% respectively. Under such decision-making paradigms, family members or patients are more inclined to make independent medical decisions without relying on the doctor's recommendations.

### Binary logistic regression outcomes

This study employed a binary logistic regression model to empirically analyze the factors influencing the medical decision-making patterns (Unilateral Decision-making and Collaborative Decision-making) among Chinese residents. The detailed regression outcomes are presented in Table 3.

Based on the results presented in Table 4, the analysis revealed several significant predictors within the realms of individual characteristics, family characteristics, occupational characteristics, and medical insurance characteristics. Regarding individual characteristics, gender emerged as a significant factor, with males (β = 0.206, *p* < 0.05) more likely to engage in unilateral decision-making, as indicated by an odds ratio (OR) of 1.228. This suggests that gender plays a crucial role in decision-making styles. Additionally, education level was inversely related to unilateral decision-making (β = -0.113, *p* < 0.05), with an OR of 0.893, implying that higher education levels may foster a more collaborative approach. Furthermore, the presence of religious beliefs was positively associated with unilateral decision-making (β = 0.237, *p* < 0.05; OR = 1.267), suggesting that religious convictions might influence individual decision-making tendencies.

**Table 4:** Binary logistic regression outcomes

Factor	Variable	Regression Coefficient	Standard Error	Wald χ2	Odds Ratio (OR)	95% Confidence Interval (CI) for OR
Individual Characteristics	Gender: Male (Reference Group: Female)	0.206* (2.243)	0.092	5.031	1.228	1.026 ~ 1.470
	Education Level	-0.113* (-1.988)	0.057	3.950	0.893	0.800 ~ 0.998
	Age	0.163 (1.469)	0.111	2.157	1.177	0.947 ~ 1.462
	Religious Belief: Yes (Reference Group: No)	0.237* (2.182)	0.108	4.762	1.267	1.024 ~ 1.567
Family Characteristics (Reference Group: Unmarried)	Family Status_2.0	0.307* (2.467)	0.124	6.086	1.359	1.065 ~ 1.734
	Family Status_3.0	0.172 (0.618)	0.279	0.382	1.188	0.688 ~ 2.052
	Family Status_4.0	0.065 (0.366)	0.177	0.134	1.067	0.754 ~ 1.511
	Family Status_5.0	-0.220 (-1.774)	0.124	3.146	0.802	0.629 ~ 1.023
	Family Status_6.0	-0.479 (-1.771)	0.270	3.135	0.620	0.365 ~ 1.053
	Family Status_7.0	-0.385 (-1.169)	0.330	1.366	0.680	0.356 ~ 1.298
	Family Status_8.0	-0.796 (-1.243)	0.640	1.546	0.451	0.129 ~ 1.582
	Monthly Family Income	-0.029 (-0.667)	0.043	0.444	0.972	0.893 ~ 1.057
Occupational Characteristics (Reference Group: Unemployed)	Occupation_1.0	1.101** (4.261)	0.258	18.153	3.008	1.812 ~ 4.992
	Occupation_2.0	0.473 (1.923)	0.246	3.700	1.604	0.991 ~ 2.597
	Occupation_3.0	0.706** (2.719)	0.260	7.395	2.027	1.218 ~ 3.372
	Occupation_4.0	0.707** (2.592)	0.273	6.720	2.029	1.188 ~ 3.464
	Occupation_5.0	0.746* (2.278)	0.327	5.190	2.108	1.110 ~ 4.003
	Occupation_6.0	0.476 (1.050)	0.454	1.102	1.610	0.662 ~ 3.919
	Occupation_7.0	0.276 (1.028)	0.269	1.058	1.318	0.779 ~ 2.232
	Occupation_8.0	0.102 (0.384)	0.267	0.147	1.108	0.656 ~ 1.871
	Occupation_9.0	0.401 (1.636)	0.245	2.678	1.493	0.924 ~ 2.412
Medical Insurance Characteristics (Reference Group: Fully Self-Paid)	Rural Residents' Basic Medical Insurance	-0.503** (-3.805)	0.132	14.478	0.605	0.466 ~ 0.783
	Urban Residents' Basic Medical Insurance	-0.193 (-1.818)	0.106	3.306	0.825	0.670 ~ 1.015
	Occupational Basic Medical Insurance	-0.333** (-3.412)	0.098	11.639	0.716	0.592 ~ 0.868
	Commercial Insurance	0.261* (2.394)	0.109	5.730	1.299	1.048 ~ 1.608
	Publicly Funded Medical Care	0.236* (2.345)	0.101	5.497	1.266	1.039 ~ 1.542
Regional Characteristics (Reference Group: Western Region)	Central	-0.074 (-0.505)	0.146	0.255	0.929	0.698 ~ 1.236
	Eastern	0.075 (0.624)	0.120	0.390	1.078	0.852 ~ 1.365
	Intercept	-1.182** (-3.733)	0.317	13.935	0.307	0.165 ~ 0.570

Dependent Variable: Decision-making Category; Unilateral Decision-making set as the dummy variable (value = 1), with Collaborative Decision-making as the reference category (value = 0)

McFadden's R-squared: 0.065

^*^ *p*<0.05 ** *p*<0.01 (z-values in parentheses)

In the domain of family characteristics, the status labeled as Family Status_2.0 was significantly associated with unilateral decision-making (β = 0.307, *p* < 0.05; OR = 1.359), highlighting the influence of specific familial contexts on decision-making processes. Occupational characteristics also played a significant role, with certain occupations (Occupation_1.0, 3.0, 4.0, 5.0) demonstrating a higher propensity for unilateral decision-making compared to unemployment. This is evidenced by relatively high odds ratios (3.008, 2.027, 2.029, and 2.108, respectively), suggesting that occupational roles can significantly shape decision-making preferences. Medical insurance characteristics further influenced decision-making styles. Individuals with Rural Residents' Basic Medical Insurance or Occupational Basic Medical Insurance were less likely to engage in unilateral decision-making (ORs = 0.605 and 0.716, respectively), as opposed to those with Commercial Insurance or Publicly Funded Medical Care, who were more inclined towards unilateral decision-making (ORs = 1.299 and 1.266, respectively).

The model's intercept was significantly negative (β = -1.182, *p* < 0.01; OR = 0.307), indicating a generally low propensity for unilateral decision-making when all explanatory variables are at their reference levels. Notably, certain factors such as age, various family statuses, and regional characteristics (Central and Eastern) did not exhibit significant impacts on the decision-making category. This lack of significance suggests that these factors may not play a substantial role in determining decision-making styles within the context of this study.

The goodness-of-fit for the model, as indicated by McFadden's R-squared, was 0.065. Although this value might appear modest, it is within the acceptable range for logistic regression models in social science research, where perfect fit is rare due to the complexity of human behaviors and decision-making processes. The value of 0.065 suggests that while the model captured key aspects influencing medical decision-making, there are other unmeasured variables and inherent complexities in the data that are not fully explained by the model. This highlights the multifaceted nature of medical decision-making and the need for further research to explore additional influencing factors.

## Discussion

### Distinctive trends in China's medical decision-making models within a cultural context

The study's analysis of decision-making patterns in China offers a unique perspective, contrasting notably with prevalent trends in Western healthcare systems. In unilateral decision-making, the prominent 'Doctor-dominant' model, accounting for 23.22% of responses, reflects a cultural preference for physician authority, diverging from Western emphasis on patient autonomy [43, 47]. This inclination towards physician-led decisions aligns with traditional values of respecting authority figures, a theme recurrent in Chinese society [2].

In collaborative decision-making, the 'Doctor-Patient-Family' model, representing 24.44%, highlights the integral role of family in medical decisions. This triadic approach is deeply rooted in China's family-centric culture [20] and contrasts with the Western focus on individual patient autonomy in Shared Decision Making [6]. The 'Doctor-led' model's prevalence (29.90%) further emphasizes the deference to medical expertise, underscoring cultural differences in patient engagement and trust in healthcare professionals [29, 31].

This research elucidates a paradigmatic shift in medical decision-making models, emphasizing the cultural congruence of a triadic model within the Chinese context. The prevalence of the 'Doctor-Patient-Family' model, as delineated in the findings, resonates with the collectivist ethos and entrenched family-centric values of Chinese society. This model diverges from the predominantly dyadic frameworks of patient-physician interaction that underpin Western healthcare models, which predominantly focus on individual autonomy as per SDM principles. The incorporation of family perspectives into the decision-making process aligns with the Confucian tenets of familial piety and collective welfare, deeply embedded in Chinese culture [3, 51]. This triadic approach not only enhances the cultural appropriateness of healthcare interventions but also ensures that decision-making is reflective of a broader familial context, potentially leading to more harmonious and satisfactory healthcare outcomes. Consequently, this research contributes to a burgeoning dialogue in global health, challenging the universality of the SDM model, predominantly rooted in Western individualism. It posits that in societies like China, where familial dynamics play a pivotal role, healthcare decision-making models must evolve to incorporate these cultural specificities.

### Key factors influencing medical decision-making patterns

According to the analysis results from Table 3, different groups exhibit varied decision-making preferences when it comes to medical decision-making patterns. In terms of individual characteristics, factors such as gender, education level, and religious beliefs have a significant impact on the choice of medical decision-making models. Specifically, males and individuals with religious beliefs tend to lean more towards the unilateral decision-making model. This can be attributed to traditional cultural contexts where males or those with religious convictions often place a higher emphasis on individual decision-making autonomy [19]. Conversely, individuals with higher educational levels tend to favor the collaborative decision-making model. This inclination might stem from the belief that those with more advanced education place greater value on the collaborative relationship between doctors and patients, thinking that such collaboration can lead to better treatment outcomes [27, 32].

In terms of family characteristics, marital status has a significant influence on the choice of medical decision-making patterns. Specifically, individuals who are married but childless tend to lean more towards the unilateral decision-making model compared to their unmarried counterparts. Firstly, this inclination may be related to the fact that those who are married but without children are in the process of family planning [5], and in certain medical decision-making scenarios, they prefer this model. Secondly, unmarried individuals might be more reliant on their family and social networks when making medical decisions [1], hence their inclination towards a collaborative.

In terms of occupational characteristics, certain job categories significantly influence the choice of medical decision-making patterns. Specifically, individuals in professions such as civil servants, corporate staff, enterprise workers, self-employed and migrant workers tend to favor the unilateral decision-making model. Civil servants, in particular, display the strongest inclination towards this model. This tendency might be attributed to the nature of their jobs and their social status, as they often prioritize efficiency [14]. On the other hand, corporate staff, enterprise workers, self-employed and migrant workers might be more concerned about medical expenses and have limited access to information [53], leading them to lean more towards unilateral decision-making approach.

Regarding the characteristics of medical insurance, certain insurance categories significantly influence the choice of medical decision-making patterns. Specifically, individuals who opt for commercial insurance and publicly funded medical care tend to lean towards the unilateral decision-making model. In contrast, those who choose the rural residents' basic medical insurance and the occupational basic medical insurance for employees are more inclined towards a collaborative decision-making approach. This divergence might be attributed to the differences in medical services and the range of choices provided by different types of insurance [7].

In terms of regional characteristics, residents from the central and eastern regions did not show significant differences in their medical decision-making patterns compared to those from the western regions. Firstly, despite regional disparities in economic development, education levels, and medical resources, the fundamental values and lifestyles of residents are profoundly influenced by Chinese culture [26]. Secondly, with the advancement of information technology [48], especially the internet and mobile communication technologies, the means and speed at which residents from different regions access medical information have greatly improved [17]. Therefore, even with disparities in economic and medical resources across regions, individuals did not exhibit significant differences in their choices of medical decision-making patterns.

Medical decision-making is a complex process, involving the balancing of various factors such as treatment outcomes, costs, and potential risks. Thus, even though certain groups may have clear inclinations in their medical decisions, these tendencies are not absolute.

### Policy implications

In the Chinese medical environment, the choice of medical decision-making models is a multi-dimensional, multi-factorial complex process, closely related to individual characteristics, family background, occupational attributes, and types of medical insurance. This study provides empirical references for policymakers and medical institutions, helping to more precisely meet the medical needs of different groups. Based on the aforementioned research findings, this study proposes the following suggestions and countermeasures:

Firstly, Enhance Doctor-Patient Collaboration. Recognizing the critical role of the cooperative decision-making model, particularly the physician-led cooperative approach which aligns closely with the principles of Shared Decision Making, it becomes essential for medical institutions to prioritize training for doctors and healthcare professionals. This training should focus on the importance of effective communication with patients and their families, a cornerstone of SDM, thereby fostering a stronger partnership between doctors and patients [21, 52]. Effective communication training, central to SDM, has been shown to significantly improve patient outcomes and satisfaction. It equips healthcare professionals with the essential skills to understand and address patient concerns more effectively, encouraging patient involvement in their own care [40]. Moreover, considering the pivotal role of the family in many cultural contexts, a balanced approach, integral to SDM, should be adopted to reconcile potential conflicts between individual autonomy and family-centered decision-making [36]. Training in SDM can also enhance the understanding of cultural nuances among healthcare providers, leading to more culturally sensitive and patient-centered care, a critical aspect of SDM [46].

Secondly, Enhance Targeted Health Literacy. The study indicates that individuals with higher levels of education tend to prefer the cooperative decision-making model. This suggests that individuals with higher educational levels are generally more receptive to health literacy education, facilitating their active participation in decision-making processes [22]. Enhanced health literacy enables patients to engage more effectively in discussions about their care [9, 30]. Therefore, there is a need for increased opportunities in health education and training, particularly targeting patient groups with lower educational levels. Such focused interventions are crucial for improving collaborative decision-making in healthcare settings [33].

Thirdly, Pay Attention to Occupational and Social Role Factors. Different occupational groups have distinct preferences in medical decision-making models. Medical institutions should recognize the differentiated characteristics among various occupational groups, taking into account their social and cultural backgrounds, to offer more personalized medical services and decision support [45]. Fourthly, Refine the Medical Insurance System. The type of medical insurance has a significant impact on the choice of medical decision-making models. Policy makers should consider adjusting medical insurance policies to ensure that all types of medical insurance can provide reasonable, transparent, and efficient medical services to their beneficiaries [37].

In conclusion, medical decision-making is a complex process involving multiple factors. It requires the joint efforts of policy makers, medical institutions, and patients to ensure that patients can enjoy more humane and efficient medical services, thereby enhancing patient satisfaction.

## Conclusions

This study delves deeply into the medical decision-making patterns of Chinese residents and their core influencing factors. The results reveal that the collaborative decision-making model predominates, especially the doctor-guided collaborative model and the joint decision-making model involving doctors, patients, and family members. This finding aligns with China's cultural background and family-centric values.

Furthermore, this research uncovers several key factors that significantly impact medical decision-making patterns, including religious beliefs, family status, occupation, and medical insurance. These factors not only reflect an individual's socio-economic status but are also closely related to culture, beliefs, and social structure. Therefore, medical decision-making is a complex phenomenon influenced by a myriad of factors. To better meet the medical needs of residents, policymakers and medical practitioners should consider these factors comprehensively and adopt corresponding strategies and measures. Simultaneously, as society evolves and transforms, medical decision-making patterns might change, necessitating continuous research and attention to consistently enhance the quality of medical services.

Nevertheless, the study, insightful in its examination of medical decision-making in China, presents two notable limitations. Initially, its constrained scope, focusing predominantly on a single cultural setting, may not encapsulate the vast array of challenges and dynamics prevalent in global healthcare systems, characterized by systemic inefficiencies, heightened costs, and persistent inequalities. Future endeavors will involve exploring and contrasting medical decision-making patterns across various global healthcare settings. This expansion will allow for a more comprehensive understanding of how cultural, economic, and systemic factors universally impact patient preferences and decision-making in healthcare, providing a more holistic view aligned with the dynamic nature of global healthcare challenges. Secondly, the study's reliance on binary logistic regression might not fully unravel the complex, multi-layered nature of healthcare decision-making. Moving forward, future research aims to incorporate more sophisticated models, such as nested logistic regression, to more effectively capture the hierarchical and diverse dimensions of healthcare decision-making, thereby enriching and broadening the study's applicability and relevance in the global healthcare context.

### Supplementary Information

**Supplementary Material 1.** 

## Data Availability

The datasets used and/or analysed during the current study available from the corresponding author on reasonable request.
