# Supplementary material for: Exploring the medical decision-making patterns and influencing factors among the general Chinese public: a binary logistic regression analysis
Source: BMC Public Health. 2024 Mar 25;24:887. doi: 10.1186/s12889-024-18338-8 (PMC10962172; doi:10.1186/s12889-024-18338-8)
Supplement: Supplementary file 1 — Supplementary Material 1. [file 12889_2024_18338_MOESM1_ESM.docx]

**Appendix 1:**

**Questionnaire**

I. Basic Information

1.Your gender is: [Single choice] [Mandatory]

○ Male ○ Female

2.Your age is: [Single choice] [Mandatory]

○ 18-44 ○ 45-59

○ 60-74 ○ 75 and above

3.Your educational level is: [Single choice] [Mandatory]

○ Junior high school and below

○ High school (Vocational)

○ College

○ Bachelor's degree and above

4.Your occupation is: [Single choice] [Mandatory]

○ Civil servant

○ Corporate Staff

○ Enterprise Worker

○ Self-employed

○ Migrant worker

○ Retiree

○ Freelancer

○ Public institution employee

○ Medical institution worker

○ Unemployed

5.Your family's monthly income is: [Single choice] [Mandatory]

○ Below 5000 yuan (excluding 5000 yuan)

○ 5000-8000 yuan (excluding 8000 yuan)

○ 8000-12000 yuan (excluding 12000 yuan)

○ 12000-23000 yuan (excluding 23000 yuan)

○ 23000 yuan and above (including 23000 yuan)

Note: This income standard refers to the research results of the Boston Consulting Group (BCG) China Consumer Insight Think Tank's 2014 China Family Income Survey Report.

6.Your medical payment method is: [Multiple choice] [Mandatory]

○ Fully self-funded

○ Publicly funded medical care

○ Commercial insurance

○ Occupational Basic Medical Insurance

○ Urban Residents' Basic Medical Insurance

○ Rural Residents' Basic Medical Insurance

7.Your religious beliefs are: [Single choice] [Mandatory]

○ Yes ○ No (Atheism)

8.Your family situation is: [Single choice] [Mandatory]

○ Unmarried

○ Married without children

○ Widowed/Divorced, with children

○ Children studying or working away from home, couple living together

○ Couple living with children

○ Elderly-centered, two or more couples and their children living together

○ Grandparents living with grandchildren

○ Other family types

9.The province (city) you are in is: [Single choice] [Mandatory]

○ Anhui

○ Beijing

○ Chongqing

○ Fujian

○ Gansu

○ Guangdong

○ Guangxi

○ Guizhou

○ Hainan

○ Hebei

○ Heilongjiang

○ Henan

○ Hubei

○ Hunan

○ Jiangsu

○ Jiangxi

○ Jilin

○ Liaoning

○ Inner Mongolia

○ Ningxia

○ Qinghai

○ Shandong

○ Shanghai

○ Shanxi

○ Shaanxi

○ Sichuan

○ Tianjin

○ Xinjiang

○ Tibet

○ Yunnan

○ Zhejiang

II. Medical Decision-Making Survey

The term "medical decision-making" in this survey refers to the entire process that includes raising medical issues, clarifying diagnostic and treatment objectives, formulating diagnostic and treatment plans, and determining the best approach. The "medical decision-making subject" refers to the individual who makes the final decision on the medical plan and bears the responsibility for that decision. Which of the following medical decision-making models do you think is more reasonable? [Single choice] [Mandatory]

1. Doctor-dominant Subtype

Unilateral Decision-making Type: The doctor makes the final judgment and determines the medical plan independently based on consultation, examination, and lab results, without being influenced by the patient.

1. Doctor-led Subtype

Collaborative Decision-making Type: The doctor makes the final judgment and determines the medical plan based on consultation, examination, and lab results, and after fully communicating with the patient and considering the patient's opinions.

1. Patient-driven Subtype

Unilateral Decision-making Type: The patient independently decides whether to accept the medical plan formulated by the doctor or chooses a treatment plan based on the doctor's opinions and suggestions.

1. Family-centric Subtype

Unilateral Decision-making Type: The family member decides whether to accept the medical plan formulated by the doctor or chooses a treatment plan based on the doctor's opinions and suggestions, without seeking the patient's opinion.

1. Doctor-Patient Subtype

Collaborative Decision-making Type: The doctor and patient jointly make the final judgment and determine the medical plan based on consultation, examination, and lab results, and after fully communicating and considering the patient's opinions.

1. Patient-Family Subtype

Collaborative Decision-making Type: The patient and family member jointly decide whether to accept the medical plan formulated by the doctor or choose a treatment plan based on the doctor's opinions and suggestions.

1. Doctor-Patient-Family Subtype

Collaborative Decision-making Type: The doctor, patient, and family member jointly make the final judgment and determine the medical plan based on consultation, examination, and lab results, and after fully communicating and considering the opinions of both the patient and family member.

**Appendix 2**

Table 1. Division of the Three Major Economic Zones

| Economic Zones | Provinces/Cities |
| --- | --- |
| Eastern | Beijing, Shanghai, Guangdong, Tianjin, Shandong, Liaoning, Jiangsu, Hebei, Zhejiang, Fujian, and Hainan. |
| Central | Anhui, Jiangxi, Shanxi, Jilin, Heilongjiang, Henan, Hubei, and Hunan. |
| Western | Guangxi, Inner Mongolia, Chongqing, Sichuan, Guizhou, Yunnan, Tibet, Shaanxi, Gansu, Qinghai, Ningxia, and Xinjiang. |
